# Perilesional resection technique of glioblastoma: intraoperative ultrasound and histological findings of the resection borders in a single center experience

**DOI:** 10.1007/s11060-022-04232-z

**Published:** 2023-01-23

**Authors:** Carlo Giussani, Giorgio Carrabba, Chiara Benedetta Rui, Gaia Chiarello, Giovanni Stefanoni, Chiara Julita, Andrea De Vito, Maria Allegra Cinalli, Gianpaolo Basso, Paolo Remida, Giuseppe Citerio, Andrea Di Cristofori

**Affiliations:** 1 Department of Medicine and Surgery, School of Medicine and Surgery, University of Milano-Bicocca, Milan, Italy; 2 Neurosurgery, Fondazione IRCCS San Gerardo dei Tintori, Via Pergolesi 33, 20900 Monza, MB Italy; 3 Neuropathology, Fondazione IRCCS San Gerardo dei Tintori, Via Pergolesi 33, MB 20900 Monza, Italy; 4 Neurology, Fondazione IRCCS San Gerardo dei Tintori, Via Pergolesi 33, 20900 Monza, MB Italy; 5 Radiotherapy, Fondazione IRCCS San Gerardo dei Tintori, Via Pergolesi 33, 20900 Monza, MB Italy; 6 Neuroradiology, Fondazione IRCCS San Gerardo dei Tintori, Via Pergolesi 33, 20900 Monza, MB Italy; 7 Neurointensive Care Unit, Fondazione IRCCS San Gerardo dei Tintori, Monza, Italy

**Keywords:** Brain tumors, Neurosurgery, Glioblastoma, Extent of resection, Progression free survival, En bloc resection, Perilesional resection, Intraoperative ultrasound

## Abstract

**Introduction:**

The surgical goal in glioblastoma treatment is the maximal safe resection of the tumor. Currently the lack of consensus on surgical technique opens different approaches. This study describes the “perilesional technique” and its outcomes in terms of the extent of resection, progression free survival and overall survival.

**Methods:**

Patients included (n = 40) received a diagnosis of glioblastoma and underwent surgery using the perilesional dissection technique at “San Gerardo Hospital”between 2018 and 2021. The tumor core was progressively isolated using a circumferential movement, healthy brain margins were protected with Cottonoid patties in a “shingles on the roof” fashion, then the tumorwas removed en bloc. Intraoperative ultrasound (iOUS) was used and at least 1 bioptic sample of “healthy” margin of the resection was collected and analyzed. The extent of resection was quantified. Extent of surgical resection (EOR) and progression free survival (PFS)were safety endpoints of the procedure.

**Results:**

Thirty-four patients (85%) received a gross total resection(GTR) while 3 (7.5%) patients received a sub-total resection (STR), and 3 (7.5%) a partial resection (PR). The mean post-operative residual volume was 1.44 cm^3^ (range 0–15.9 cm^3^).During surgery, a total of 76 margins were collected: 51 (67.1%) were tumor free, 25 (32.9%) were infiltrated. The median PFS was 13.4 months, 15.3 in the GTR group and 9.6 months in the STR-PR group.

**Conclusions:**

Perilesional resection is an efficient technique which aims to bring the surgeon to a safe environment, carefully reaching the “healthy” brain before removing the tumoren bloc. This technique can achieve excellent tumor margins, extent of resection, and preservation of apatient’s functions.

**Supplementary Information:**

The online version contains supplementary material available at 10.1007/s11060-022-04232-z.

## Introduction

Glioblastoma (GB) is a primary brain tumor with an infiltrative behavior that may affect both eloquent or non-eloquent areas of the brain [1–4]. Current standard of care for GB involves tumor resection followed by adjuvant chemo-radiation therapy according to the Stupp protocol [5]. Gross total resection (GTR) of the contrast-enhancing part of the tumor is known to influence survival outcome of patients affected by this disease, while the benefits and extent of supramarginal resection are still under debate [6–9].

Owing to tumor location and its infiltrative nature, and despite several tools being available to optimize the extent of resection (EOR) such asneuronavigation, neurophysiological monitoring, intra-operative imaging, microscopic fluorescence etc. [6, 10, 11], it is known that it could be difficult in some situations to achieve a safe, complete resection of the contrast-enhancing part of the glioblastoma.

This fact is related to the infiltrative subcortical nature of this disease that often does not have a cleavage plane that allows a separation of the tumor from the surrounding brain parenchyma. Until now, while surgery of extra-axial lesions aims to find the dissection plane in-between the brain and the tumor, in the case of intra-axial tumors two main approaches can be performed: central debulking and perilesional dissection [12–14].

In GBs, the first rule is to avoid any neurological deficit that can lead to the worsening of the prognosis of the patient [15–17]. As a consequence, some neurosurgeons approach GBs with an intralesional debulking approach that aims to gradually remove the tumor with a piecemeal technique [13]. This technique comes from the idea that beginning the resection in the middle of the tumor and centrifugally widening the aspiration would be a safe approach to enable the surgeon to stop when normal appearing white matter is reached. Other surgeons may face GBs starting from the tumor boundaries with a perilesional resection; a technique in which the GB is resected in an enbloc fashion [12, 13]. In this way, the surgeon has to figure out a cleavage plane that goes around the tumor while the contrast-enhancing bit of the tumor is left intact. According to a recent work by Sawaya and Colleagues, perilesional resection of GBs leads to a higher rate of GTRs and to a lower rate of neurological complications than in intralesional debulking. The increased overall survival reported may be related to a more effective surgical cytoreduction than to the piecemeal technique [12].

In this work, we aimed to describe in detail the perilesional resection technique and to prospectively analyze the histological findings of the surgical margins of a series of 40 consecutive patients with a histopathological diagnosis of glioblastoma (WHO 2021), who underwent surgery at our institution in order to better investigate the benefits of en bloc resection with perilesional dissection.

## Materials and methods

### Patient selection

In this study we prospectively included 40 patients aged more than 18 years old operated for GB resection from 2019 to 2020 at *Azienda Socio Sanitaria Territoriale Monza—Ospedale San Gerardo Monza*, Italy. Patients who received a different diagnosis to IDH wild-type GB were excluded. All surgeries were performed using the perilesional dissection technique with intraoperative ultrasound (iOUS) and neuronavigation. When surgical resection was considered complete by the surgeon, at least one bioptic sample from clean tumor cavities were collected and sent to the pathology department.

Data collection was performed prospectively and encompassed demographic data including age at diagnosis, sex, side and site of the tumor, neurological deficits at presentation and/or after surgery, overall survival (OS) and progression free survival (PFS). Follow-up ended on 30th September 2022.

Extent of surgical resection (EOR) was measured according to Berger et al. 2011 based on pre-operative and post-operative volumetric T1-weighted with gadolinium brain MRIs [18]. GTR was considered when 99% of the tumor volume was removed; while STR was considered when tumor was removed from 98 to 80% and PR when below 80%.

Informed consent was obtained from all individual participants included in the study.

### Surgical technique and sample collection

**Fig. 1:**
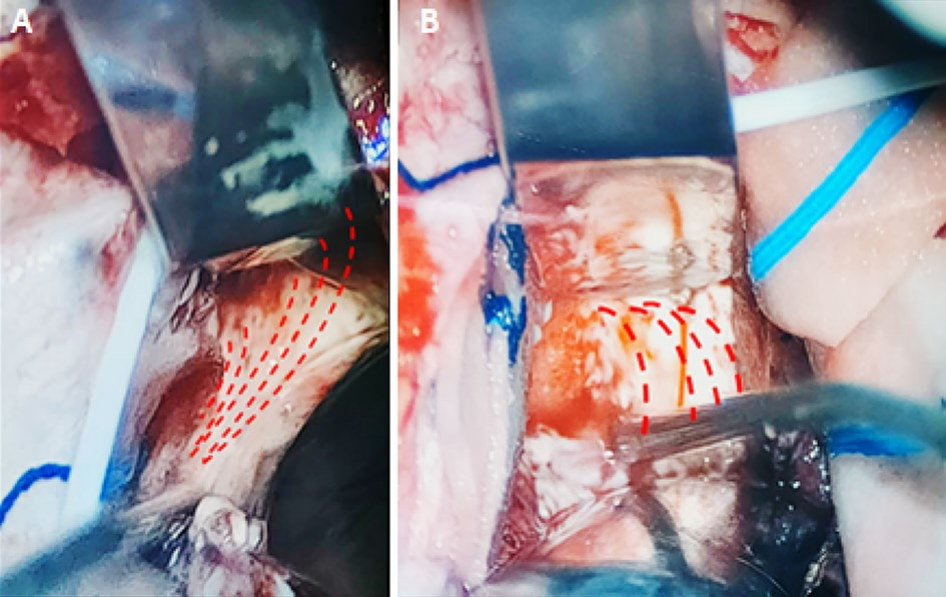
Shows the identification of a perilesional plane through traction with dedicated dissection spatulas. A and B show a case of a left parietal GB

A tailored craniotomy with the aid of neuronavigation is performed. After durotomy and exposure of the brain, a corticectomy around the most superficial part of the tumor is performed. A sort of perilesional plane around the contrast-enhancing part of the tumor is found through the tractionof the tumor away from the normal appearing brain using dedicated spatulas (see Fig. 1).

**Fig. 2:**
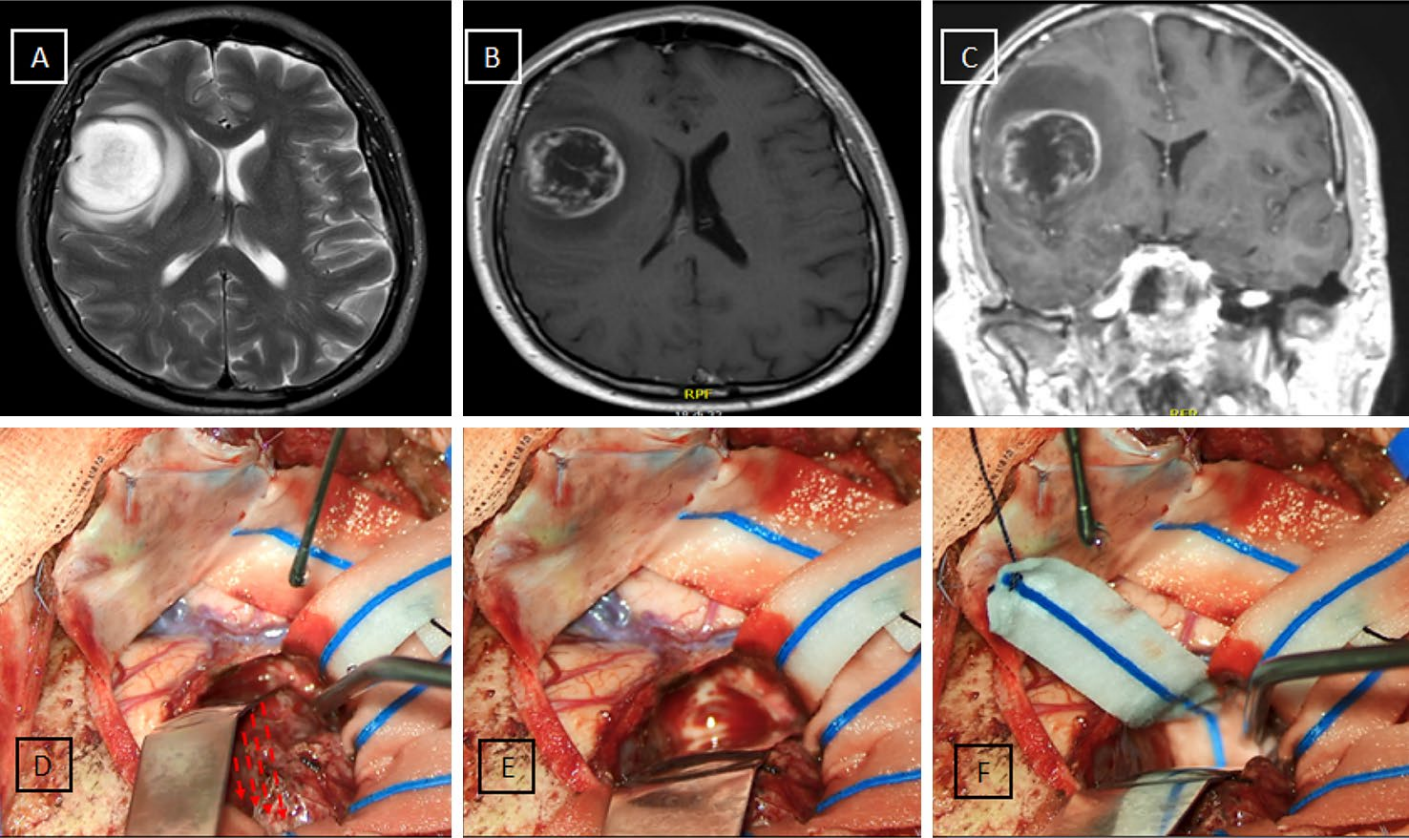
Shows the circumnavigation of a case of a right fronto-opercular GB: **A**T2-weighted preoperative MRI; **B**,**C** T1-weighted with gadolinium pre-operative brain MRI. **D**–**F** A dissection plane is found and delimited with cottonoid patties

In this way, the white matter is gradually and circumferentially suctioned until the bottom of the tumor is reached. This circumferential movement allows the surgeon to perform the hemostasis during the surgical resection progressively;it also delineates a separation plane in the white matter (or at the level of the arachnoid sulci when they form a part of the dissection planes) that can be protected placing a cottonoid pattie over another in a “shingles on the roof” fashion while the circumferential dissection is progressing. The use of neuronavigation and intraoperative ultrasound (iOUS) reduces the risk of losing a correct trajectory around the tumor; in this way, this is circumnavigated (see Fig. 2).

**Fig. 3:**
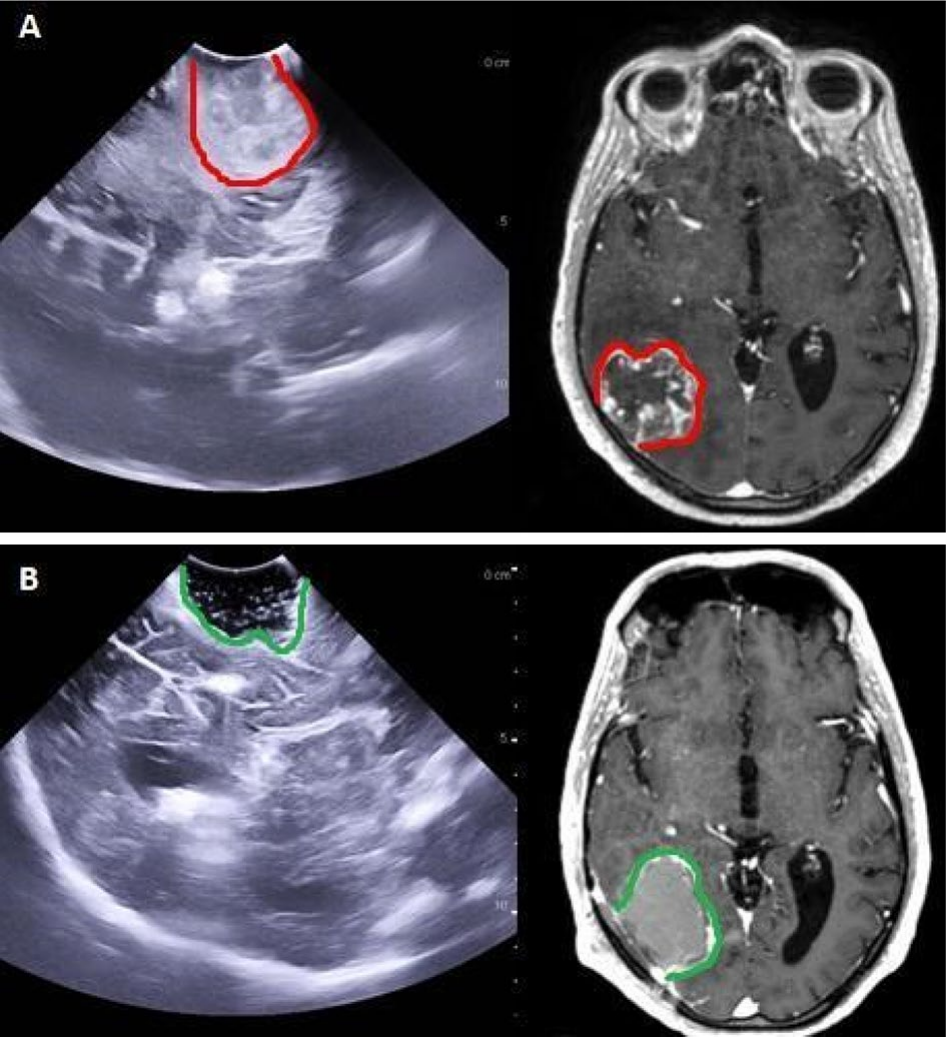
This figure shows a case of a right temporal GB operated with the perilesional dissection technique. In this picture it is possible to understand how US and MRI can look like similar pictures

After the bottom is reached, a cottonoid pattie is placed over the normal appearing white matter. When the tumor has been circumnavigated, it can be detached from the bottom of the surgical cavity and removed enbloc with a film of normal-appearing white matter attached to the contrast-enhancing tumor. When the volume of the tumor mass does not allow the surgeon to spatulate the white matter, it can be centrally debulked, like in meningioma surgery, to permit tractions (see Supplemental Fig. 1). Hemostasis is easily performed with gentle retraction of the patties and coagulating the strips of white matter attached to them. An online intraoperative video is available in the supplementary materials. After resection is considered completed by the primary surgeon and checked with an iOUS, biopsy samples are randomly collected with dedicated forceps from the walls of the surgical cavity that are not considered in relation to eloquent areas (see Fig. 3). No surgical adjuncts are used in order to avoid false negative results.

### Histology

**Fig. 4:**
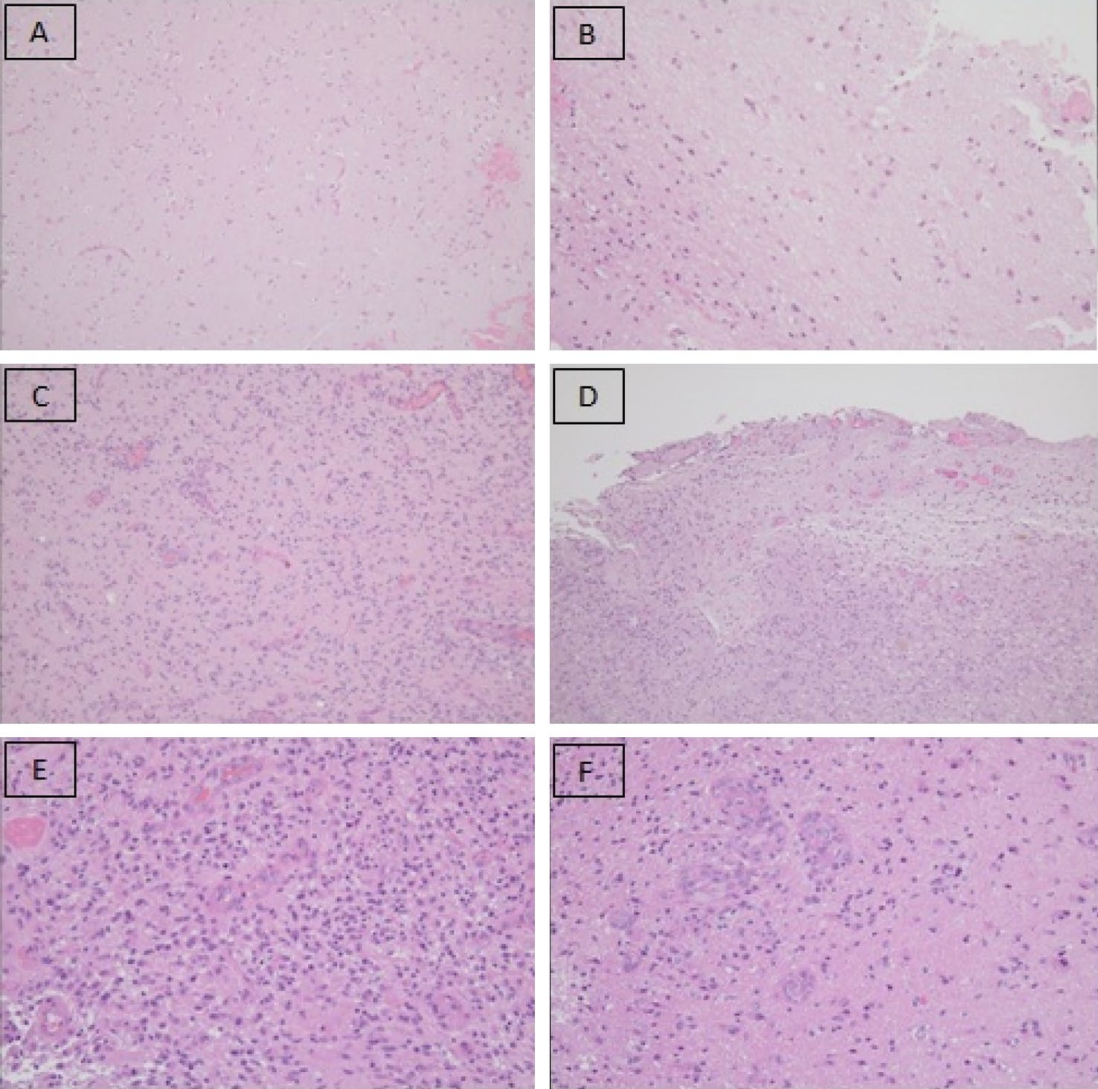
Example of histological findings of perilesional biopsies. **A**-**B** normal appearing brain; **C**-**D** infiltration of perilesional brain; **E**-**F** frankly tumoral perilesional tissue

Histological diagnosis was performed by a dedicated pathologist blinded to the intraoperative and to post-operative MRI findings. Diagnosis of GB was reviewed in accordance with the 2021 WHO criteria [3]. Routine screenings for IDH mutation, LOH and MGMT status were performed. Perilesional samples were considered free of tumor when no tumoral cells wereidentified; infiltrated when increased cellularity and atypical cells were found; and tumoral when they resembled the histological findings of the tumor core diagnosis. In Fig. 4 the kind of histological findings on perilesional biopsies are reported (see Fig. 4).

## Results

In our series we included 40 patients, 26 males and 14 females with a median age of 66 years, affected by IDH wild type GBs. MGMT was found to be hypermethylated in 15 patients while none of the patients expressed LOH of 1p19q nor a mutation of IDH. Pre-operative median KPS was 90 and ranged from 40 to 100. Pre-operative neurological deficits were present in 18 patients. Tumors were left-sided in 18 cases and locations included 4 parietal lesions, 16 temporal lesions, 14 frontal lesions, 5 occipital lesions and 1 insular lesion. All patients were operated with a circumferential perilesional technique by the senior author (C. G.) as described in Methods.

Pre-operative tumor volume, in terms of contrast-enhancing lesion, ranged from 1.5 to 96.4 cm^3^. Thirty-four patients (85%) received a GTR while 3 (7.5%) patients received a sub-total resection (STR) and 3 (7.5%) patients received a partial resection (PR). Mean post-operative residual volume was 1.44 cm^3^ (range 0–15.9 cm^3^).

During surgery, a total of 76 margins were collected from the surgical bed as described in Methods. Of the 76 samples, 51 (67.1%) were tumor free (like in Fig. 4 A, B) and 25 (32.9%) were infiltrated by the tumor (like in Fig. 4C, D). No frankly tumoral samples (like in Fig. 4E, F) were isolated. Seventy-one margins were collected among patients that received a GTR: 21 (29.6%) appeared infiltrated by glioma and 50 (70.4%) were tumor free.

Post-operative complications were present in 5 cases: one patient developed expressive aphasia, two patients developed a left-sided hemiparesis, one patient developed right-sided hemiparesis and one patient developed left-sided hemiplegia. Permanent neurological deficits were reported in one patient, while others recovered before starting adjuvant treatments.

Among patients that received a GTR, 16/34 (51.6%) were alive at the end of the follow-up without recurrence. The median OS of all patients was 12.7 months; the median OS in patients that received a GTR was 19.3 months, while the median OS in patients without a GTR was 8.6 months and all of these patients were dead from tumor progression at the end of the follow-up. The median PFS was 13.4 months; in particular 15 months in the GTR group and 9.6 months in the STR-PR group.

## Discussion

In our work we described the histological findings on the surgical cavity of patients undergoing surgical resection of GB with a specific technique: the perilesional resection. This way of removing an intra-axial tumor resembles a sort of enbloc resection that is usually performed during oncological surgery in other districts of the human body [12].The technique described in our paper is a way of applying principles of general oncology to neurological surgery and, given the low incidence of post-operative neurological deficits described in our series, perilesional resection can be considered a safe way of approaching a GB.

This technique was first described by Sawaia et al. in [12]. In their work, a huge cohort of patients with GB operated with either central debulking or perilesional dissection was described. In particular, they demonstrated that enbloc resection of GB was associated with a better overall survival and with a higher rate of GTR [12].

Starting from the work by Sawaia [12] and colleagues, we wanted to understand those findings from a biological point of view. In our cohort of patients, 76 margins from 40 patients were histologically analyzed with 50 samples with no tumor. All patients with tumor free histological samples had received a GTR and none of them had a post-operative deficit. These findings can be considered very interesting when compared with those found in some works reported in literature with a similar numbers of patients [19, 20]. Only few reports are available in literature about histology from tumoral margins with variable results. The work by Kubben et al. [21] described the histological characterization of39 biopsies collected on the borders of the surgical cavities of 10 patients with the aid of intraoperative MRI. In their work all biopsy samples were characterized by the presence of an infiltrative tumor that could sometimes resemble a lower grade glioma [21]. Other works report absence of tumoral cells in the perilesional samples collected [22, 23]. In the work by Mangiola [22] and colleagues, no tumoral cells were seen in the peritumoral margins taken far from the tumor core except in a small sample of patients [22, 23]. Their findings may suggest that the region of sampling may be of importance for histological results. Another work was published by Eidel et al. in 2017 with a similar number of patients enrolled in our study [20]. During their study, they collected samples from stereotactic biopsies from both contrast-enhancing and non-enhancing parts of GBs. They found that the non-contrast-enhancing parts had the highest relative content of viable tumor cells [20]; but this may reflect the fact that tumors selected for stereotactic biopsy might be more diffuse and highly infiltrative ones than tumors eligible for surgical resection.

In a recent work by Coburger et al. using iOUS, 5-ALA and intraoperative MRI,only 1 in 33 patientsafter an assumed GTR had perilesional margins free of tumor [19]. In their work it is not clear which strategy was used to surgically remove a GB, whether by perilesional or intralesional resection or both.

Such differences between our findings and the findings reported in literature may be related to different factors: the surgical technique used and the concomitant reduction of tumoral cells density in the peripheral zone of the GB (as reported by Mangiola in 2012) [22]. The small size of bioptic samples retrieved at the end of surgery may be another factor affecting the results of our study although the size of such samples can be compared to a common stereotactic biopsy sample.

**Fig. 5:**
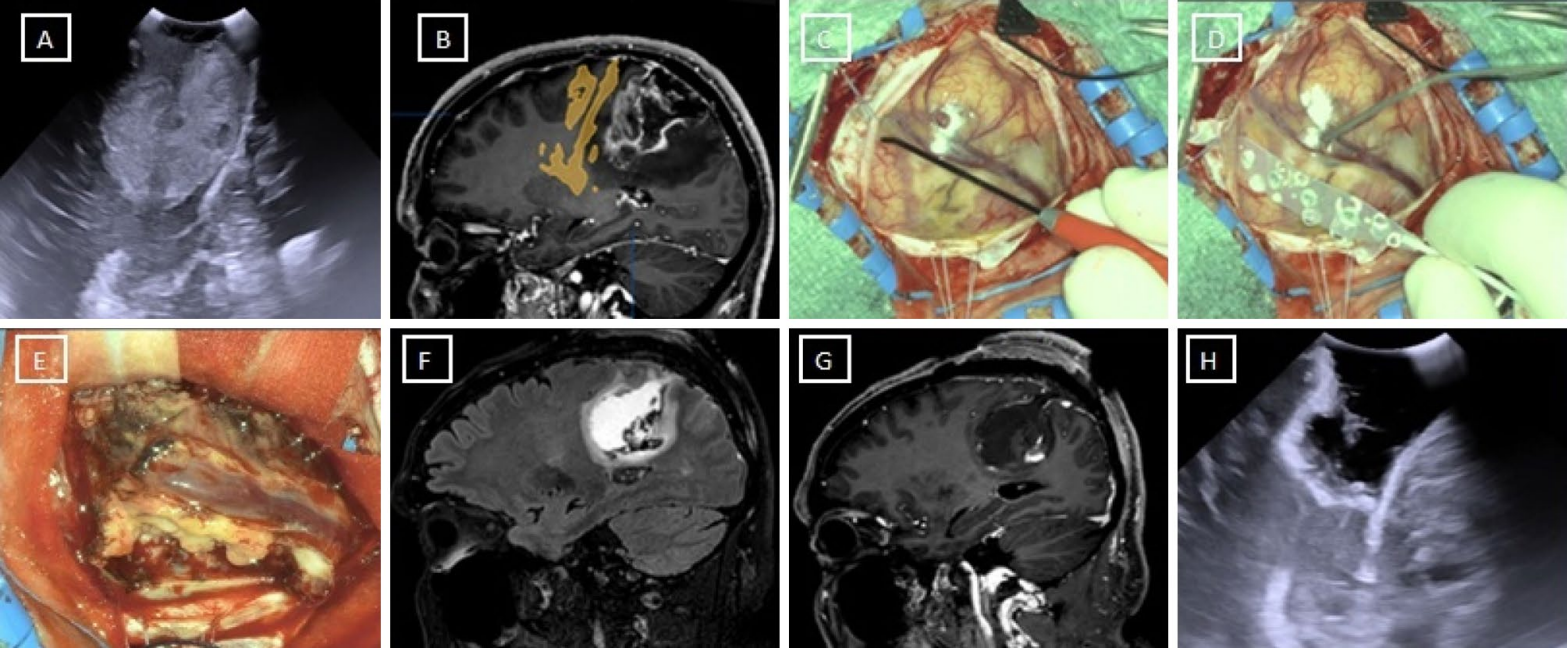
Example of perilesional dissection technique for resection of a perirolandic tumor. The Corticospinal tract had been identified with a monopolar probe and continuously monitored with a cortical motor strip during resection (**B**)

In a speculative way, surgical technique may influence the findings of the results on biopsies on the perilesional tissue. If we consider the study by Eidel: they performed stereotactic biopsies on patients with GBs in the border of the tumor at the passage between necrotic, contrast-enhancing and non-enhancing zones [20]. Their approach was not resective, andas a consequence may deliver a detailed histological description of the peritumoral zone which is not present in studies where the peritumoral zone is delineated after a cytoreductive surgery. In fact, in the last scenario it may be difficult to recognize the peritumoral zoneas it may not lookthe same as in the pre-operative MRI scans sinceits extension and location is modified (e.g. a brain shift during tumor resection [24, 25]) and it undergoes reshaping due to surgery. Moreover, as reported by several authors and also in our series, in GB it is not always possible to achieve a GTR since the tumor is not always easy to distinguish from the apparently healthy brain. As a consequence, the surgical approach for resection may lead to different histological results on the biopsy sampled. In fact, the perilesional resection may guide the surgeon to start the glioma resection from “healthy” brain tissue with no neoplastic cellular density rather than starting the resection from frankly tumoral areas. As described in the intraoperative images, surgical resection aims to remove the tumor without looking at its borders (see Fig. 5) and it can be considered a sort of supramarginal resection. In this way, in the case of perilesional resection, the peritumoral zone with viable tumoral cells may be removed during the dissection of the tumor from the peritumoral brain; while in the case of central debulking, the peritumoral zone is resected by the surgeon in a centrifugal way and as a consequence, peritumoral tissue with viable tumoral cells may be left behind. As a consequence, bioptic samples may be tumor free with the first technique and tumor infiltrated with the second technique.

From a functional and morphological perspective, GBs tend to form a bulk that displaces white matter bundles rather than infiltrating them [26]. In this view, central debulking can lead to the start of the surgical resection from the less eloquent area of the surgical cavity towards the more potentially eloquent area of the surgical cavity. In this way, it is possible to expose the most eloquent field of the surgical cavity at the end of the procedure.

On the other hand, taking advantage of the tendency of GBs to displace white matter bundles as described in DTI studies [26], perilesional resection of the tumor does not lead to resection of white matter bundles unless they are very close to the tumor. This is why this technique resulted as safe in our series with a low rate of post-operative deficits. Moreover, accurate surgical planning with tractography can lead us to better understand and estimate the distance between the tumor and the functionally intact white matter. Starting from the findings at the pre-operative MRI, it is possible to plan what white matter zones are at risk of surgical damage. Moreover, intraoperative neurophysiological monitoring can also help to preserve eloquent white matter bundles and to define the distances between the tumor bulk and the white matter tract to be preserved (see Fig. 5). In particular, in order to preserve motor function during the perilesional resection, we found Raabe’s technique with dynamic mapping of corticospinal tract [27] useful.

Taking into consideration the findings ofiOUS in patients that received a GTR, it was possible to see that the iOUS was able to confirm histologically tumor free margins in about 70% of cases and a perilesional infiltrating front in about 30% of cases. In none of these cases a frankly tumoral zone was found. This finding confirmed that US is a good intraoperative tool to check for tumor remnants, as proposed in pediatric brain tumor surgery [28]. In our series, 6 patients received a STR-PR due to highly extensive GB or due to a very close proximity to eloquent white matter bundles that were at risk of intraoperative injury even with resection under neurophysiological monitoring.

*Limits of the perilesional technique* The perilesional approach shows two challenging difficulties: first, the surgeon has to find a boundary plane between the contrast-enhancing (CE) tumor and the perilesional “healthy” brain; second, the surgeon has to plan the surgery carefully so as to avoid damage of eloquent white matter tracts that may be displaced by the tumor in order to avoid a resection of the perilesional plane with functionally important white matter tracts. Moreover, in some cases, this approach may not be feasible due to tumor location. For example, in the case of purely insular GBs, it may be difficult to find a safe perilesional plane due to the vicinity of the tumor to the sylvian fissure and the small perforating branches of the middle cerebral artery; or it may be difficult in the case of thalamic gliomas due to the involvement of deep white matter bundles. In these cases, it may be possible to mix the two techniques (central debulking or perilesional resection) in accordance with the brain anatomy. From the perspective of biopsy sampling, our study is in contrast with the results of other similar studies, although this may be related tothe site of sampling and with the small sample size of the biopsy.

Even if tumor resection may be completed with a perilesional technique, GB still has a poor prognosis due to a high recurrence rate;in any case, some encouraging data regarding the benefits of supramarginal resection of GBs have been published [9, 29]. Taking into account the paper by Molinaro et al. about supramarginal resection [9], enbloc resection of GBs might increase the chances to obtain an extensive supramarginal resection since this technique may allow the quick removal of the contrast-enhancing part of the tumor as a first surgical step. At the same time, it allows the exposure of the remaining perilesional tissue which may be removed in a second surgical step with ultrasonic aspirator, iOUS and intraoperative neurophisiological monitoring in order to functionally navigate through “healthy” white matter and to prevent the risk of neurological deficits. Moreover, analyzing the results by Molinaro et al. elderly patients (over 65 years of age) do not seem to benefit from the supramarginal resection making the perilesional resection a good surgical option [9].On the other hand, according to the findings published by the group of Quinones-Hinojosa, the EOR of the non-CE part of the tumor can improve survival even if it is less important than the EOR of the CE part of the tumor [30].

### Limits of the study

Our study has some limitations that encompass the small number of patients included. Moreover, our results are in contrast with other publications, and this might be related to the size of the perilesional biopsy sample taken. The selection of the biopsy site was another bias of our study although a random selection of the biopsy site was the main policy when the surgical bed was considered not adjacent to eloquent areas. Biopsy selection site can be considered a bias per se since sampling could not be performed in the same way for all patients but it needed to be tailored in accordance with tumor location. In any case, the absence of neoplastic cells in the majority of the samples may indicate that the sampling was performed far away from the central core.

## Conclusion

The perilesional resection of GBs is an efficient technique which aims to bring the surgeon to a safe environment, reaching the “healthy” brain carefully before removing the tumoren bloc. This technique is safe, and can lead to good tumor margins, a good rate of extent of resection, and the preservation of a patient’s functions with low rate of neurological deficits and complications. Moreover, in the oncological view of maximizing the saferesection of GBs beyond the CE tumor boundaries, this technique can efficiently allow the neurosurgeon to quickly expose the infiltrative FLAIR hyperintense part of the tumor.

## Supplementary Information

Below is the link to the electronic supplementary material.

Supplementary file1 (MP4 923993 KB)

Supplementary file2 (PNG 490 KB)

## Data Availability

The datasets generated during and/or analysed during the current study are available from the corresponding author on reasonable request.
