# Supplementary figures and images for: Perilesional resection technique of glioblastoma: intraoperative ultrasound and histological findings of the resection borders in a single center experience
Source: J Neurooncol. 2023 Jan 23;161(3):625–32. doi: 10.1007/s11060-022-04232-z (PMC9992251; doi:10.1007/s11060-022-04232-z)

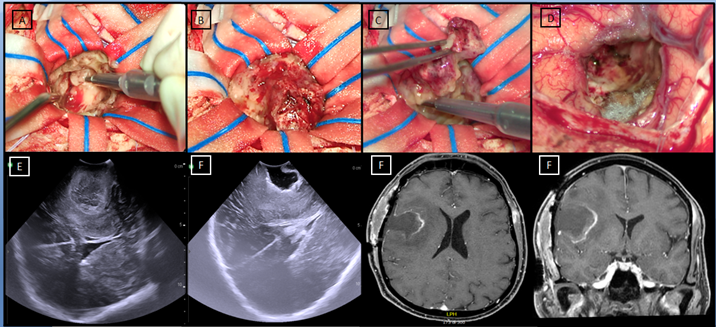

Supplement: Supplementary file 2 — Supplementary file2 (PNG 490 KB) [file 11060_2022_4232_MOESM2_ESM.png]
